# Fatty acid and lipidomic data in normal and tumor colon tissues of rats fed diets with and without fish oil

**DOI:** 10.1016/j.dib.2017.06.032

**Published:** 2017-06-23

**Authors:** Zora Djuric, Muhammad Nadeem Aslam, Becky R. Simon, Ananda Sen, Yan Jiang, Jianwei Ren, Rena Chan, Tanu Soni, Thekkelnaycke M. Rajendiran, William L. Smith, Dean E. Brenner

**Affiliations:** a Department of Family Medicine, University of Michigan, Ann Arbor, MI 48109, United States; b Department of Pathology, University of Michigan, Ann Arbor, MI 48109, United States; c Department of Internal Medicine, University of Michigan, Ann Arbor, MI 48109, United States; d Departments of Statistics, University of Michigan, Ann Arbor, MI 48109, United States; e Michigan Metabolomics Resource Center, University of Michigan, Ann Arbor, MI 48109, United States; f Department of Biochemistry, University of Michigan, Ann Arbor, MI 48109, United States; g Department of Pharmacology, University of Michigan, Ann Arbor, MI 48109, United States

**Keywords:** Fatty acids, Colon tumorigenesis, Diet, Fish oils, Lipidomics

## Abstract

Data is provided to show the detailed fatty acid and lipidomic composition of normal and tumor rat colon tissues. Rats were fed either a Western fat diet or a fish oil diet, and half the rats from each diet group were treated with chemical carcinogens that induce colon cancer (azoxymethane and dextran sodium sulfate). The data show total fatty acid profiles of sera and of all the colon tissues, namely normal tissue from control rats and both normal and tumor tissues from carcinogen-treated rats, as obtained by gas chromatography with mass spectral detection. Data from lipidomic analyses of a representative subset of the colon tissue samples is also shown in heat maps generated from hierarchical cluster analysis. These data display the utility lipidomic analyses to enhance the interpretation of dietary feeding studies aimed at cancer prevention and support the findings published in the companion paper (Effects of fish oil supplementation on prostaglandins in normal and tumor colon tissue: modulation by the lipogenic phenotype of colon tumors, Djuric et al., 2017 [1]).

**Specifications Table**

**Table:** 

Subject area	Biology
More specific subject area	Cancer Biology
Type of data	Tables and heat maps
How data was acquired	Two methods of lipid analysis were employed: Gas-chromatography with mass spectral detection (GC–MS) and liquid chromatography with tandem mass spectral detection (LC–MS–MS).
Data format	Means and standard deviations, results of hierarchical analysis in heat maps
Experimental factors	Rats were fed either a Western fat diet or a fish oil diet. Half the rats in each diet group were treated with a chemical carcinogen to induce colon tumors.
Experimental features	Male F344 rats were placed on the experimental diets at 5 weeks of age followed by carcinogen treatment at 6–7 weeks of age (azoxymethane and dextran sodium sulfate). Rats were sacrificed at 26 weeks of age. Normal and tumor colon tissues were flash frozen prior to extraction and analysis. Lipid extraction was done by the Folch method prior to total fatty acid analyses by GC–MS. Lipid extraction of select colon tissues for lipidomic analyses was done using the Bligh–Dyer method.
Data source location	University of Michigan, Ann Arbor, MI
Data accessibility	The data is shown in the Tables and Figure. It will also be available six months after article publication at http://www.metabolomicsworkbench.org/data/browse.php
Related research article	Effects of fish oil supplementation on prostaglandins in normal and tumor colon tissue: modulation by the lipogenic phenotype of colon tumors. Zora Djuric, Muhammad Nadeem Aslam, Becky R. Simon, Ananda Sen, Yan Jiang, Jianwei Ren, Rena Chan, Tanu Soni, Thekkelnaycke M. Rajendiran, William L. Smith and Dean E. Brenner; J. Nutr. Biochemistry, 46 (2017): 90–99.

**Value of the data**

• The data show fatty acids and lipids in four groups of rats: rats randomized to receive a fish oil or Western diet, and a chemical carcinogen or not.
• The complete fatty acids profiles by gas chromatography of total lipid extracts are provided.
• The lipidomic data show specific lipids in three types of colon tissues: normal tissue from control rats, normal tissue from carcinogen treated rats and tumor tissue from carcinogen treated rats.
• Lipidomic profiles also show the effects of a fish oil diet in normal and tumor colon tissues.

## 1 Data

**Fig. 1:**
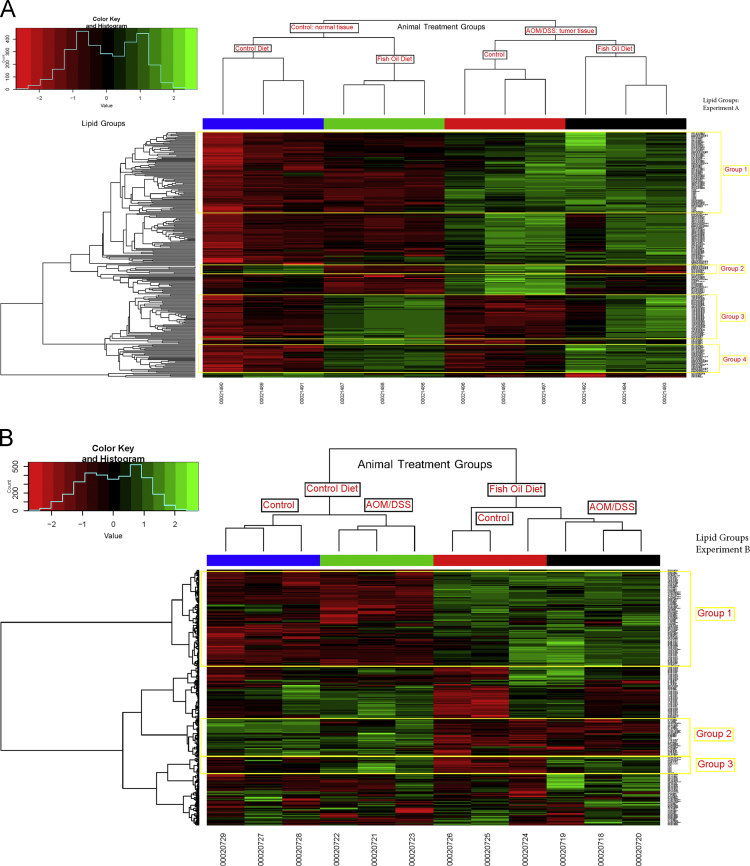
Lipidomic analysis of distal colon tissue. Shown are the results of hierarchical cluster analysis of lipids that differed significantly by carcinogen treatment or diet type, after correction for false discovery rates. The lipid groups for Experiment A refer to a lipidomic analysis of tumor tissues from carcinogen-treated rats and normal tissues from control rats. The rats in either case were fed either a Western fat or fish oil diet. Lipid groups for Experiment B refer to lipidomic analysis of normal tissues from control rats and normal tissues from carcinogen-treated rats. The annotated lipid groups highlight those lipids that were altered by either 1) the fish oil diet versus the Western fat diet consistently in all samples across tissue type or to 2) those lipids that were consistently altered by tissue type without regard to diet. Abbreviations: cardiolipin, CL; diglycerides, DG; free fatty acids, FFA; monoglycerides, MG; phosphatidylcholine, PC; phosphatidylethanolamine, PE; PG; phosphatidylinositol, PI; phosphatidylserine, PS; Sphingomyelin, SM; triglycerides, TG. Interpretation of these results is shown in the accompany research paper [1]. **A**. Normal colon tissue from control rats and tumor tissue from rats treated with carcinogen. In each group, rats were fed either the Western fat (control) or fish oil diet. Lipids that differed significantly by group, after correction for false discovery rates, are shown. Legend: Blue, normal colon from control rats fed the Western fat diet; Green, normal colon from control rats fed the fish oil diet; Red, tumor tissue from carcinogen-treated rats fed the Western fat diet; and Black, tumor tissue from carcinogen-treated rats fed the fish oil diet. **B**. Grossly normal colon tissue from rats treated with carcinogen (AOM and DSS) or from control rats, fed either the Western fat (control) or fish oil diet. Legend: Blue, normal colon from control rats fed the Western fat diet; Green, normal colon from carcinogen-treated rats fed the Western fat diet; Red, normal colon from control rats fed the fish oil diet; Black, normal colon from carcinogen-treated rats fed the fish oil diet.

Data tables are provided for individual fatty acids obtained from gas chromatographic analysis of fatty acid methyl esters for serum (Table 1) and for colon tissues (Table 2). The related research article [1] does not report all the fatty acids singly. In addition, a subset of the tissue samples was analyzed by lipidomics and the heat maps of all the lipids that were identified by hierarchical cluster analyses to differ by tissue type or diet type are shown in Fig. 1.

**Table 1:** Individual serum fatty acids and fatty acid ratios used to estimate desaturase activity. Data shown is mean and SD, and starred values are significantly different by diet within each treatment group (carcinogen treatment or not) by two-sample *t*-tests using a cut-off of *p*≤0.001. There were 10 rats per group. ANOVA of fatty acid classes is shown in the accompany research paper [1].

Fatty acid (mole % or mole ratio)	Serum of control rats		Serum of carcinogen-treated rats	
	Western fat diet	Fish oil diet	Western fat diet	Fish oil diet
12:0	2.62 (0.25)	3.23 (0.79)	3.19 (0.87)	3.37 (0.65)
14:0	2.64 (0.25)	2.98 (0.52)	2.94 (0.45)	2.96 (0.36)
16:0	27.12 (1.62)	26.72 (1.95)	25.75 (1.58)	25.74 (1.79)
16:1	2.08 (0.41)	2.59 (0.34)	1.54 (0.55)	2.07 (0.41)
18:0	10.78 (1.05)	10.86 (1.00)	11.03 (0.94)	12.04 (0.77)
18:1	23.88 (1.37)	15.71 (1.53)*	23.39 (1.51)	14.47 (1.02)*
18:2, ω-6	11.58 (0.58)	10.45 (0.83)	12.46 (1.39)	11.03 (0.33)
18:3, ω-3	0.31 (0.03)	0.52 (0.06)*	0.34 (0.06)	0.47 (0.04)*
20:1	0.31 (0.02)	0.19 (0.04)*	0.31 (0.02)	0.19 (0.03)*
20:3, ω-3-6	0.57 (0.05)	0.81 (0.12)*	0.51 (0.06)	0.88 (0.06)*
20:4, ω-6	14.12 (1.65)	7.37 (1.39)*	14.55 (1.90)	7.96 (0.82)*
20:5, ω-3	0.23 (0.05)	7.00 (0.70)*	0.22 (0.06)	6.85 (0.83)*
22:4, ω-6	1.09 (0.19)	0.40 (0.11)*	1.09 (0.19)	0.40 (0.11)*
22:5, ω-3	0.71 (0.11)	3.34 (0.53)*	0.71 (0.12)	3.34 (0.53)*
22:6, ω-3	1.95 (0.34)	7.84 (1.49)*	2.02 (0.29)	8.19 (0.96)*
Ratio 18:1–18:0	2.24 (0.32)	1.47 (0.24)*	2.14 (0.29)	1.21 (0.15)*
Ratio 16:1–16:0	0.08 (0.01)	0.10 (0.01)*	0.06 (0.02)	0.08 (0.01)

**Table 2:** Fatty acids in distal colon of rats fed different diets. Data shown is mean and SD, and starred values are significantly different by diet within each treatment group (carcinogen treatment or not) by two-sample *t*-tests using a cut-off of *p*≤0.001. There were 10 rats per group and 9 per group for tumor tissue from carcinogen-treated rats fed the fish oil diet. ANOVA of fatty acid classes is shown in the accompany research paper [1].

Fatty acid (mole %, or mole ratio)	Normal tissue from control rats		Normal tissue from carcinogen-treated rats		Tumor tissue from carcinogen-treated rats	
	Western fat diet	Fish oil diet	Western fat diet	Fish oil diet	Western fat diet	Fish oil diet
12:0	5.47 (1.85)	4.73 (0.78)	6.73 (1.42)	5.82 (1.19)	4.06 (1.78)	6.14 (2.51)
14:0	4.69 (0.68)	4.84 (1.26)	4.83 (0.82)	4.91 (0.62)	3.72 (0.91)	3.91 (1.23)
16:0	19.11 (1.61)	19.00 (2.51)	18.95 (1.40)	20.26 (2.15)	21.11 (1.23)	20.88 (2.49)
16:1	5.74 (1.68)	9.78 (1.68)*	4.91 (1.85)	8.99 (3.02)	2.90 (1.02)	4.32 (1.50)
18:0	6.90 (3.41)	5.76 (2.20)	5.16 (2.13)	6.60 (2.94)	11.46 (2.10)	9.75 (3.24)
18:1	33.52 (6.90)	29.28 (2.93)*	36.95 (5.43)	27.00 (4.45)*	24.1 (3.71)	21.6 (5.6)
18:2, ω-6	13.31 (1.62)	13.44 (0.72)	14.09 (0.94)	12.60 (0.96)	11.1 (0.96)	12.2 (1.00)
18:3, ω-3	0.52 (0.13)	0.80 (0.12)*	0.51 (0.16)	0.73 (0.15)	0.33 (0.19)	0.68 (0.20)*
20:1	0.44 (0.24)	0.36 (0.21)	0.32 (0.19)	0.37 (0.15)	1.78 (0.63)	0.66 (0.30)
20:3, ω-3-6	0.62 (0.34)	0.70 (0.33)	0.56 (0.31)	0.76 (0.33)	0.62 (0.28)	1.75 (0.50)
20:4, ω-6 (AA)	5.98 (3.61)	4.16 (2.08)	3.82 (2.26)	4.14 (1.79)	12.6 (3.7)	7.18 (3.23)
20:5, ω-3 (EPA)	1.45 (0.80)	2.61 (1.03)	1.34 (1.31)	3.31 (1.65)	2.12 (1.48)	5.19 (2.74)
22:4 ω-6 (DTA)	0.93 (0.52)	0.41 (0.25)	0.66 (0.37)	0.38 (0.16)	1.78 (0.63)	0.66 (0.30)*
22:5, ω-3 (DPA)	0.41 (0.22)	1.44 (0.51)*	0.37 (0.28)	1.38 (0.28)*	0.62 (0.28)	1.75 (0.50)*
22:6, ω-3 (DHA)	0.91 (0.58)	2.69 (0.54)*	0.82 (0.58)	2.73 (0.54)*	1.6 (0.6)	3.4 (1.0)*
Ratio 18:1–18:0	6.25 (3.34)	5.98 (2.77)	8.53 (3.84)	5.27 (3.27)	2.22 (0.74)	2.85 (2.28)
Ratio 16:1–16:0	0.31 (0.11)	0.53 (0.17)	0.26 (0.11)	0.46 (0.18)	0.14 (0.05)	0.21 (0.06)

## 2 Experimental design, materials and methods

### 2.1 Animals and diets

Male Fisher F344 rats were utilized as described in the companion paper [1]. The protocol was approved by the University Committee on Use and Care of Animals at the University of Michigan and complied with guidelines set forth by the Association for Assessment and Accreditation of Laboratory Animal Care.

Rats at six weeks of age were randomized into two dietary groups: a control group (20 rats) and a fish oil group (20 rats). Both diets contained 34% energy from fat, 17% by weight. This fat content was achieved by decreasing cornstarch in the standard AIN93G diet. The Western fat diet contained 45% coconut oil, 30% olive oil, 15% corn oil and an 10% soybean oil (by weight). The Western fat blend was mixed with menhaden oil to achieve an EPA:ω-6 ratio of 0.4 for the fish oil diet as previously described [2]. This resulted in a “fish oil” diet that was 6.2% Menhaden oil, as percent of the total diet. The diets were prepared by Dyets Inc. (Bethlehem, PA) and stored at −80 °C [1]. Half of the rats in each diet group were administered 15 mg/kg body weight of azoxymethane by intra-peritoneal injection one week after starting the experimental diets followed by 2% dextran sodium sulfate in the drinking water for seven days.

Rats were sacrificed at 26 weeks of age using carbon dioxide inhalation. Blood was obtained using cardiac puncture and sera were stored at −80 °C for fatty acid analysis. The colon was quickly removed, opened longitudinally and rinsed with ice-cold phosphate-buffered saline (PBS) containing indomethacin (5.6 μg/mL). Tumors in the distal colon were dissected away from normal tissue before snap freezing in liquid nitrogen. The colon tissues were subsequently pulverized in liquid nitrogen and stored at −80 °C.

### 2.2 Fatty acid analysis by GC–MS

A portion of the pulverized colon tissue was used for analysis of fatty acids and protein as previously described [1]. Briefly, a homogenate was prepared and extracted with Folch reagent. Fatty acid methyl esters (FAME) were prepared using 0.2 N methanolic (m-trifluoromethylphenyl) trimethylammonium hydroxide and GC–MS was conducted using selected ion monitoring [3]. The weight amount of each fatty acid (in µg) was calculated from standard curves and converted to moles. For serum samples, the mole percentage of each fatty acid was calculated, of total fatty acids. For colon samples, µmoles of each fatty acid per µg protein were calculated as well as the mole percent of each fatty acid. Data was stratified by tissue type and two-sample *t*-tests were conducted to evaluate the diet type effect.

### 2.3 Lipidomics of colon tissue

Two separate, untargeted, shotgun lipidomic experiments were carried out using aliquots of the pulverized, flash-frozen colon tissue samples as previously described [1]. The lipidomic results were normalized to tissue weight. The first experiment compared included normal colon tissue from control rats fed fish oil or not and tumor colon tissue from carcinogen-treated rats fed fish oil or not (Fig. 1A). Three samples from each of these four treatment/diet groups were selected based on their colonic prostaglandin E_2_ (PGE_2_) concentrations to represent maximize diversity in the samples [1]. A second lipidomic experiment included normal colon tissue from control rats and grossly normal colon tissue from rats treated with carcinogen, with three rats in each treatment group fed either the Western diet or the fish oil diet (Fig. 1B). The samples again were selected to represent maximum diversity in colonic PGE_2_ in the samples.

Extraction of lipids from pulverized tissue was carried out after spiking with internal and sonicating the samples in methanol. Lipids were extracted using water: methanol: dichloromethane (1:1:1) and analyzed by HPLC–MS–MS. Lipids were identified using the LIPIDBLAST library and were quantified by normalizing against internal standards. After adjusting for false discovery rates, those lipids with *p*<0.05 for the effect of diet or colon tissue type were subjected to hierarchical clustering analyses with the R program [4]. Heatmaps were created using the function “heatmap.2” in package “gplots” within the R program [5]. The heatmaps revealed groups of lipids that differed by diet regardless of carcinogen treatment or tissue type as well as groups of lipids that differed by tissue type regardless of diet. These lipid groups are annotated on Fig. 1.
