# Supplementary material for: Fatty acid and lipidomic data in normal and tumor colon tissues of rats fed diets with and without fish oil
Source: Data Brief. 2017 Jun 23;13:661–6. doi: 10.1016/j.dib.2017.06.032 (PMC5503825; doi:10.1016/j.dib.2017.06.032)
Supplement: Supplementary file 1 — Supplementary material [file mmc1.docx]

**
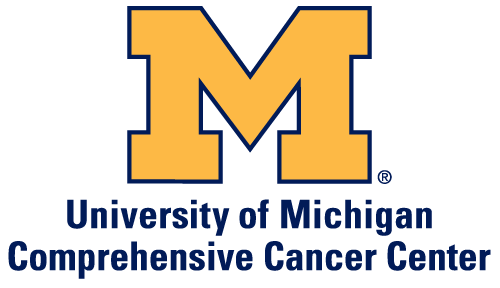
**

**Department of Family Medicine**

**University of Michigan**

**Ann Arbor, MI 48109-5930**

***Phone (734) 647-1417* Administrative Assistant**

***Phone (734) 998-7120* Research Offices**

***Fax (734) 647-9817***

May 29, 2017

The authors declare no conflicts of interest with the paper (DIB-D-17-00362, Fatty Acid and Lipodomic Data in Normal and Tumor Colon Tissue of Rats Fed Diets with and without Fish Oil”

Signed on behalf of all the authors:


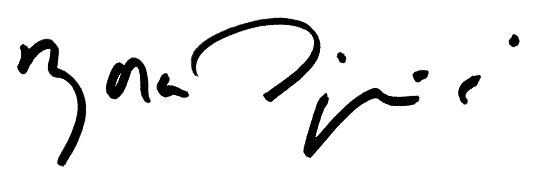


Zora Djuric, Ph.D.

Corresponding author

Research Professor of Family Medicine and

Nutritional Sciences

University of Michigan

Ann Arbor, MI, USA 48109-5930

Phone (734)615-6210

zoralong@umich.edu

Format reviewer comments:

I recommend revisions to the format and content of the manuscript to conform to the Data in Brief requirements.

* Please ensure that you have provided, within the article or in a public repository, new original DATA that have not been published elsewhere

**Reply:** The data shown are for individual fatty acids. In the J. Nutr. Biochemistry paper, summary variables are shown (eg. “12:0, 14:0, 16:0 and 18:0” versus “saturated fatty acids”, in the Data in Brief and J. Nutr. Biochem. Papers, respectively).

* The data presented in this article are required to be accessible either within the article or in a public repository. The accessibility of the data should be stated in the Specifications table

**Reply:** The Specifications Table states that the data is available in the Tables and Figure.

* Ensure that the title of your article clearly states that your article contains data

**Reply:** The title indicates that the article contains “Fatty Acid and Lipodomic Data…”

* The Data article should be purely descriptive (i.e., no results, conclusions or insightful observations about the data)

**Reply:** This has been checked. The abstract was revised and shortened. Two bullets points under “Value of the Data” are re-phrased. Instead of stating “The lipidomic data identify differences in specific lipids…” we now phrase this to “The lipidomic data show specific lipids …” Instead of “ Fish oil diets had specific effects on lipidomic profiles in normal and tumor colon tissues” we now use “Lipidomic profiles in normal and tumor colon tissues from animals fed control or fish oil diets are shown”. In addition, we found a typo that was corrected (lipodomic should be lipidomic).

* Ensure that you focus your article on the description of the data provided in this article

**Reply:** This has been checked and corrected as stated under the previous point.

* Ensure that there is no duplication of data (Figures, Table etc.) with original article or any previously published article. Duplication in another academic journal is not permitted. Delete duplications, revise and cite original references as appropriate. Please also ensure that there is no excessive duplication of text.

**Reply:** The Figures and Tables are not duplicated. The text has been revised to shorten it substantially.

* Revise your manuscript throughout accordingly to Data in Brief format as shown in this example <http://www.sciencedirect.com/science/article/pii/S2352340916305285>. For more information on the Do's and Don't to write a good Data in Brief article, see <http://media.journals.elsevier.com/content/files/datainbrief-18083024.pdf>

**Reply:** This has been checked. We have added section numbering.

* Check that all abbreviations and acronyms are spelled out

**Reply:** We have added this for one acronym that was previously omitted. Thank you for pointing this out.

* Check the text for clarity, grammar and syntax throughout. Before resubmitting your manuscript, please have a native speaker or a proofreader checking your manuscript for grammar, style and syntax.

**Reply:** We have proofread the manuscript one more time. Dr. Djuric is a native English speaker who was raised in the state of Texas. She finds this comment presumptuous and was offended by it.

* Check that all instruction texts provided in the Data in Brief template are removed.

**Reply:** Instruction texts were removed.

* The abstract (usually up to 250 words) should include reference to original article if the Data in Brief article is transferred from a research journal. This should be in abbreviated form: Title and reference number or DOI and reference number

**Reply:** Instead of referring to the “in press” article, we now give the citation since it was published recently (ref. #1).

* The "Value of the data" and "Data" sections should provide concise and written descriptions.

**Reply:** These sections are very brief.

* Value of the data

[Provide 3-5 bullet points and word each point so it describes how the data in this article (rather than the results) could be valuable to the scientific community, usually up to 250 words.]

**Reply:** This is 90 words in 4 bullet points.

* Data

[Description of the data in a concise paragraph (5 sentences or up to 250 words)]

**Reply:** This is 82 words in one paragraph.

* Experimental Design, Materials and Methods usually ~300 words

**Reply:** This is now 705 words, shortened from 1312 words.

* References

If your article was transferred to Data in Brief via another journal, ensure that you have cited and included in the reference list your main research article.

Format references according to Data in Brief guidelines and as shown below

[1] Cleveland BM, Weber GM. Effects of steroid treatment on growth, nutrient partitioning, and expression of genes related to growth and nutrient metabolism in adult triploid rainbow trout (Oncorhynchus mykiss). Domest Anim Endocrinol. 2016 56:1-12.

**Reply:** We have updated the ref #1 of the main research article that is now published. We have removed the parenthesis around the year for each reference even though the example paper uses the format that we had in the first submission.

Reviewers (if applicable):

MS language need to be corrected. English and use of grammar should appropriate. Avoid use of fancy words...

**Reply:** We are not sure what the term “fancy words” refers to. The manuscript is written in correct U.S. English at a Flesch-Kincaid readability level of grade 11.1. This is not overly complicated or “fancy” for a scientific manuscript geared towards a college-level audience. We have proofread it again and did break up a few of the longer sentences into two shorter sentences. We also shortened the methods section.
